# Oestrous cycle-dependent equine uterine immune response to induced infectious endometritis

**DOI:** 10.1186/s13567-016-0398-x

**Published:** 2016-11-08

**Authors:** Christina D. Marth, Simon M. Firestone, Lisa Y. Glenton, Glenn F. Browning, Neil D. Young, Natali Krekeler

**Affiliations:** 1 Asia-Pacific Centre for Animal Health, Faculty of Veterinary and Agricultural Sciences, The University of Melbourne, Parkville, VIC Australia; 2 Translational Research and Animal Clinical Trial Study Group, Faculty of Veterinary and Agricultural Sciences, The University of Melbourne, Werribee, VIC Australia; 3 Faculty of Veterinary and Agricultural Sciences, The University of Melbourne, Parkville, VIC Australia

## Abstract

Infectious endometritis is a major cause of reduced pregnancy rates in horses. The objectives of this study were to establish a timeline of the innate immune response in the uterus of healthy horses and to investigate the oestrous cycle effect on this. Endometrial biopsies were collected from five horses before and at 3, 12, 24, 48 and 72 h after inoculation of *Escherichia coli*, once in oestrus and once in dioestrus. They were analysed by quantitative real-time PCR, microbiology and histology. Neutrophil numbers increased from very low levels in the absence of inflammation to severe neutrophilia 3 h after inoculation. The concentrations of mRNAs for Toll-like receptor (TLR)2, TLR4, NOD-like receptor NLRC5, tissue inhibitor of metallopeptidases 1 (TIMP1) and chemokines CCL2, CXCL9, CXCL10 and CXCL11 were all increased 3 h after inoculation of *E. coli* compared to levels detected prior to inoculation. Chemokine mRNA levels remained elevated for 48 h. Concentrations of mRNAs for the antimicrobial peptides equine β-defensin 1 (EBD1), lysozyme, secretory leukoprotease inhibitor (SLPI), lipocalin 2 (LCN2), lactoferrin and uteroferrin were increased between 3 and 12 h post inoculation. The gene for secreted phospholipase A_2_ (sPLA_2_) was expressed constitutively. P19 uterocalin mRNA levels were higher in dioestrus than in oestrus over the first 24 h of inflammation. Neutrophils and many innate immune genes responded rapidly to the introduction of *E. coli* into the uterus, while the oestrous cycle stage had only a relatively minor effect on the response to *E. coli*. This study has delineated a useful model of innate immunity in infectious endometritis of healthy animals.

**Electronic supplementary material:**

The online version of this article (doi:10.1186/s13567-016-0398-x) contains supplementary material, which is available to authorized users.

## Introduction

Approximately 15% of all Thoroughbred broodmares show signs of persistent mating-induced endometritis (PMIE), a local inflammation of the superficial layers of the uterus after breeding [1]. This has a significant impact on the horse breeding industry because of its effect on pregnancy rates in affected mares [2].

Both natural breeding and artificial insemination induce a transient physiological endometritis to clear the uterus of introduced spermatozoa, seminal plasma, bacteria and cell debris [3, 4]. This contamination induces an inflammatory response with neutrophilia and increased mRNA levels of several genes associated with the innate immune response [5, 6]. Similarly, the introduction of bacteria into the uterine environment has also been shown to cause neutrophilia and increased mRNA levels of several “innate immune” genes [7].

Bacterial uterine infections are found in 25–60% of barren mares [8, 9], with the two most commonly isolated bacteria, *Escherichia coli* (*E. coli*) and *Streptococcus equi* subspecies *zooepidemicus* (*S. equi* ss *zooepidemicus*) both regarded as opportunistic pathogens [10]. While PMIE is an inflammatory response to semen, mares susceptible and resistant to PMIE have also been shown to react differently to an intrauterine inoculation of bacteria [7]. This knowledge has been used in multiple studies, analysing infectious endometritis to investigate differences between healthy mares and those susceptible to PMIE [11–13].

Furthermore, studies have suggested that the efficiency of the uterine immune response is affected by steroid hormones and that the uterus faces different challenges depending on the stage of the oestrous cycle [14, 15]. A previous next-generation sequencing study found that several pathways related to the immune response were enriched in the oestrus gene expression profiles of cycling mares when compared to their dioestrus profiles, suggesting increased immunological vigilance during oestrus [16]. Inoculation of *E. coli* into the uterus of healthy mares during oestrus [7] results in a much less pronounced inflammation than in dioestrus [17], in which purulent or serohaemorrhagic vaginal discharge, heavy bacterial growth and acute systemic inflammation were detected. A high-throughput sequencing study identified 1500 genes with significant differences when directly comparing samples taken 3 h after inoculation of *E. coli* in oestrus with those taken at the same time point in the same horses in dioestrus [18]. Overall, these studies suggest that hormone levels affect the immune response in the equine uterus.

As susceptibility to PMIE depends on differences within the first few hours after breeding, the most important factors involved in this immune response are likely to be part of the innate immune response. To gain an in-depth understanding of the innate immune cascade in the equine uterus, a next generation sequencing analysis was performed on samples taken from the equine uterus before and 3 h after inoculation of healthy mares with *E. coli*. It identified a number of immune response genes that were up-regulated after the introduction of *E. coli*, particularly in the pathogen recognition receptor (PRR) family, the chemokine and cytokine families and the antimicrobial peptide group [18].

The presence of bacteria in the body is detected by PRRs such as the Toll-like receptors (TLRs) and nucleotide-binding oligomerisation domain (NOD) receptors (NLRs). TLRs have been detected in the reproductive tracts of several mammals, including horses [19]. TLR4 is the primary receptor responsible for the detection of lipopolysaccharide (LPS), a conserved pathogen-associated molecular pattern (PAMP) of Gram-negative bacteria [20, 21], while TLR2 may have a supporting function in detecting LPS [22]. NLRs are responsible for the intracellular detection of pathogens and can activate pro-inflammatory cytokines [23].

Another important group of factors that participate in the innate immune response are the chemokines, the main function of which is the regulation of transendothelial leucocyte migration [24]. In addition, an antimicrobial potential has been identified for CXCL9, 10 and 11 [25, 26].

The endometrium, like other mucous membranes, also uses antimicrobial peptides in its defence against bacteria. They can destabilise bacterial cell walls, interfere with bacterial enzymes or inhibit bacterial iron acquisition and have been detected in multiple organs in the horse [27]. Several defensins have been shown to disrupt the integrity of the bacterial cell wall by pore formation, while lysozyme uses the hydrolysis of peptidoglycans for a similar effect [28]. Secreted phospholipase A_2_ (sPLA_2_) plays a comparable antibacterial role [29]. Secretory leukoprotease inhibitor (SLPI), which is homolog to equine neutrophil antimicrobial peptide 2 (eNAP-2) [30], selectively inhibits microbial serine proteases [31, 32]. The binding of elements essential for microbial metabolism, such as iron, achieves bacteriostasis [28]. This mechanism is used by lipocalin 2 (LCN2) [33] and lactoferrin (LFN) [34], which also binds to Lipid A of LPS [35]. Uteroferrin (UFN) is essential in trans-placental transport of iron in pigs and horses [36, 37], although it has not previously been implicated in the equine immune response. Another transport molecule associated with fatty acid transport in early equine pregnancy is P19 uterocalin, the production of which is stimulated by progesterone [38].

Tissue inhibitors of metalloproteinases (TIMPs) regulate matrix metalloproteinases (MMPs), which, in turn, have been shown to influence several components of the immune response, particularly chemokines [39, 40]. The ratio of MMP9 and TIMP1 has previously been correlated with neutrophil, eosinophil and lymphocyte counts during inflammatory processes in the lung [41].

The previous next-generation sequencing study performed on samples taken before and 3 h after inoculation of *E. coli* in the equine uterus revealed that several genes were up-regulated in response to the introduced bacteria. These included PRRs, as well as several antimicrobial peptides, chemokines, cytokines and MMPs [18]. The results of this RNA-Seq study were used to select a range of genes for analysis at later time points. Thus, the current study follows up on the previous high-throughput sequencing study by establishing a temporal profile of uterine innate immune responses in healthy horses up to 3 days after inoculation of *E. coli* using quantitative real-time PCR (qPCR). The objectives of this study were to evaluate a set of genes for inflammatory and antimicrobial factors as markers of equine endometritis and to investigate the effect of the oestrous cycle stage on expression of these genes.

## Materials and methods

### Selection of experimental animals

Endometrial biopsies were obtained from five Standardbred mares aged between 3 and 4 years. They were maintained on pasture at the facilities of the Faculty of Veterinary and Agricultural Sciences, The University of Melbourne, Australia. Treatments and procedures were approved by the University of Melbourne Animal Ethics Committee (Approval Number: 1112297).

For this study, clinically healthy mares were confirmed to be resistant to PMIE based on histopathological evaluation of the endometrium and by insemination with frozen stallion semen during oestrus. This reliably induces uterine inflammation [42]. There were no clinical signs of persistent inflammatory oedema or intrauterine fluid 24 h after insemination. A histopathological evaluation by two independent examiners classified samples from all five mares in category I according to Kenney and Doig’s classification [43]. The absence of any pre-existing bacterial infection in the uterus was confirmed by conventional bacteriological culture of uterine swabs collected from each mare prior to insemination.

### Preparation of *E. coli* inocula

An *E. coli* strain (EC_CM1) isolated from the reproductive tract of a mare susceptible to post-breeding endometritis was stored at −80 °C. To prepare inocula the strain was streaked onto a Mueller–Hinton agar plate and incubated for 24 h at 37 °C. A single colony was transferred to 2 mL of Mueller–Hinton broth and the broth incubated overnight at 37 °C. The overnight broth culture was diluted to achieve a final concentration of 10^5^ colony forming units (CFU) per inoculum. The inocula were kept on ice for a maximum of 1 h before use.

### Inoculation of *E. coli* and collection of endometrial tissue and swab samples

Follicle development, intrauterine fluid, development of uterine oedema and cervical and uterine tone were monitored daily by trans-rectal palpation and ultrasonographic examination to determine the stage of the oestrous cycle. Rectal temperature, heart rate and respiratory rate were monitored daily or at each sample collection.

A five tier oedema score system (E0–E4), with E0 representing the absence of any uterine oedema and E4 representing pathological inflammatory oedema, was used to evaluate uterine oedema indicative of cycle stage and/or inflammation.

**Figure 1:**
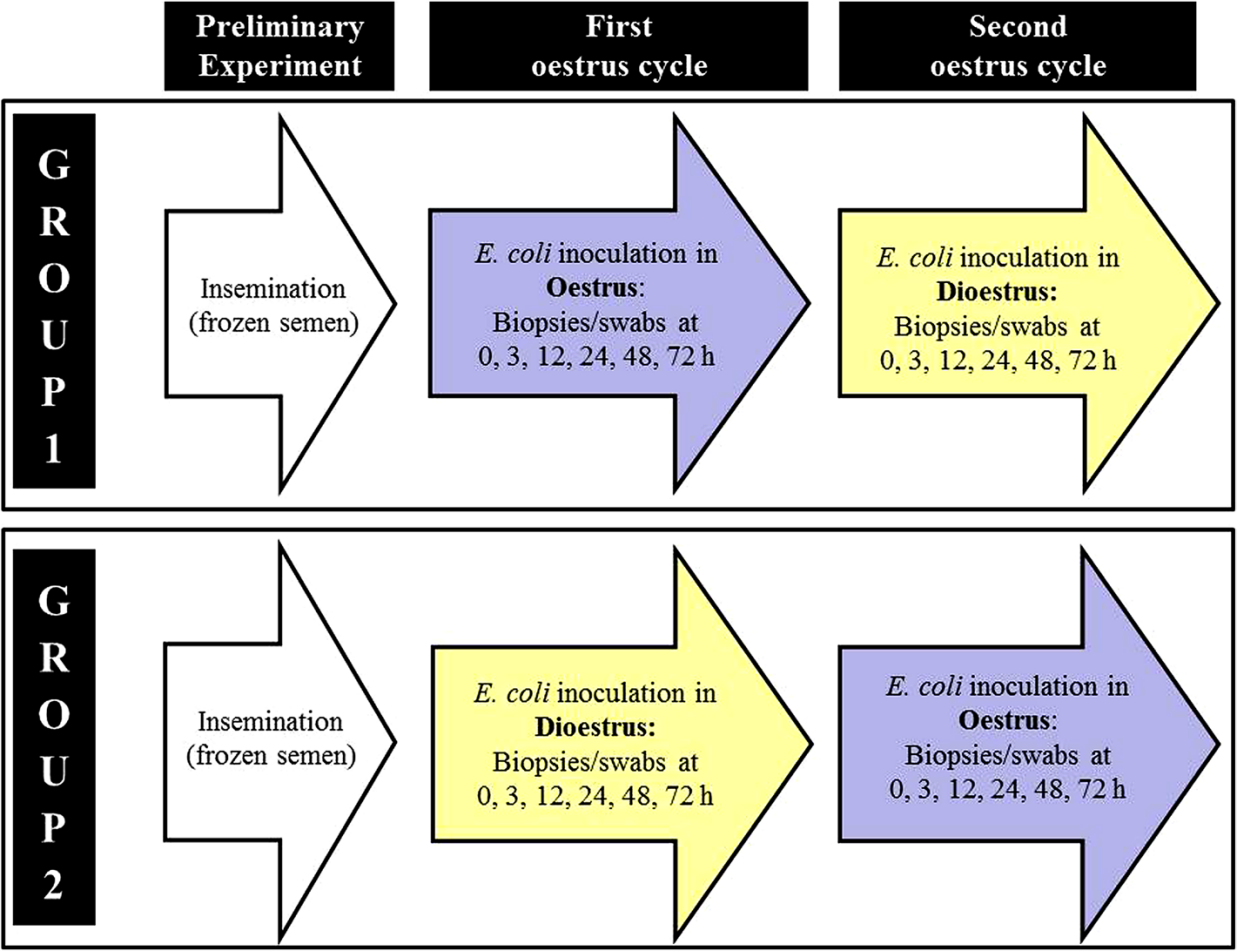
**Flow chart outlining the timeline and procedures for each experimental group.** Each experimental group consisting of two to three horses was initially inseminated with frozen semen during oestrus (preliminary experiment). Mares were then inoculated with *E. coli* in two consecutive oestrous cycles in a crossover study design. Group 1 (three mares) was first inoculated during oestrus (dominant follicle ≥35 mm, uterine oedema and decreased uterine and cervical tone) and samples were taken prior to and at several time points after inoculation. After the absence of inflammatory signs was established, they were again inoculated during dioestrus (functional corpus luteum, absence of uterine oedema and low plasma progesterone) and samples were collected at the same time points. Group 2 was first inoculated in dioestrus followed by an inoculation during oestrus. Samples were again taken at the same time points prior to and after inoculation.

Mares were inoculated with *E. coli* in two consecutive oestrous cycles in a crossover study design (Figure 1). The three randomly assigned mares in group 1 were inoculated with *E. coli* during oestrus, as indicated by the presence of a dominant follicle ≥35 mm in diameter, uterine oedema and decreased uterine and cervical tone. A sample for uterine culture was collected using a double-guarded swab (Minitube Australia, Ballarat, VIC, Australia), before endometrial tissue samples were obtained by trans-cervical biopsy before (0 h) and 3, 12, 24, 48 and 72 h post inoculation (pi) using an alligator jaw biopsy punch (Jorvet, Loveland, CO, USA). Care was taken to obtain biopsies from different sites at the base of the uterine horns alternating the left and right horn at each time point.

During the following oestrus, the absence of inflammation was established by the lack of pathological oedema, as well as the lack of neutrophilia or detectable bacterial growth. The same mares were then inoculated with the same strain of *E. coli* during dioestrus, as indicated by detection of a functional corpus luteum and the absence of uterine oedema 5 days after ovulation. In addition, serum samples were obtained to evaluate plasma progesterone prior to collection. Endometrial biopsies and swabs were again taken before (0 h) and 3, 12, 24, 48 and 72 h pi.

The two mares assigned to the second group were subjected to the same procedures in reverse order, being initially inoculated during dioestrus and subsequently during oestrus. Samples were taken at the same time points using the same methods as described above (Figure 1).

After collection, all endometrial biopsies were divided into three sections with a sterile scalpel blade. One section was used immediately for microbiological culture. One section was snap-frozen in liquid nitrogen in OCT embedding medium (Tissue-Tek, Olympus Australia Pty. Ltd, Mount Waverly, VIC, Australia) and stored at −80 °C until further processing. The final section was placed in RNAlater (Life Technologies Australia, Mulgrave, VIC, Australia) and incubated overnight on a rocking platform at room temperature before being stored at −80 °C until further processing.

### Preparation of samples for microbiology

The portion of the biopsy used for microbiological culture and the uterine swab sample were used to inoculate Mueller–Hinton-Agar plates, which were then incubated aerobically at 37 °C for 24 h to quantify and identify the bacteria in the uterus. Colony counts were scored as: no growth (<5 colony forming units (CFU)); mild growth (5–10 CFU); moderate growth (11–50 CFU); and heavy growth (>50 CFU), as described previously [44].

Representative colonies from each plate were replated onto MacConkey agar to confirm their identity as *E. coli* by assessing their capacity to ferment lactose and to produce indole from tryptophan. Results were recorded as *E. coli* or other uterine pathogen/contaminant.

### Histopathology

The OCT embedded sections of the 0, 3, 12 and 24 h samples were mounted on a cryostat (Leica, North Ryde, Australia) to cut 7–10 µm thick tissue sections, which were stained with haematoxylin and eosin (H&E). Histopathological assessments of the endometrium were made on 0 h samples according to Kenney and Doig’s classification [43].

Classification of the extent of inflammation was based on the total number of neutrophilic leukocytes present in three fields at a magnification of 400×, with the categories assigned being: no neutrophilia (<10 neutrophils in total in the three fields), moderate neutrophilia (10–99 neutrophils in total in the three fields), severe neutrophilia (100–149 neutrophils in total in the three fields) and very severe neutrophilia (>150 neutrophils in total in the three fields). In addition, the presence of neutrophils in the epithelial layer and in the endometrial glands was determined for each field and the total number of eosinophil leukocytes was counted.

### RNA extraction and cDNA synthesis

The endometrial biopsy samples placed in RNAlater were homogenised in Trizol (Qiagen, Chadstone, VIC, Australia) using a Polytron homogeniser (IKA Works, Selangor, Malaysia) and the total RNA was purified using the RNeasy Universal Plus Mini Kit (Qiagen) according to the manufacturer’s instructions. The total RNA was resuspended in 70 µL RNAse-free water and the nucleotide concentration and purity was assessed for each sample by spectrophotometry using a NanoDrop ND-1000 (Thermo Fisher Scientific Australia Pty Ltd, Scoresby, VIC, Australia). All samples had A_260_/A_280_ ratios greater than 1.99 and A_260_/A_230_ ratios greater than 1.82. RNA samples were reverse-transcribed using the iScript cDNA synthesis kit (Bio-Rad, Gladesville, Australia) according to the manufacturer’s instructions and the resulting cDNA diluted to a concentration equivalent to 2 ng total original RNA/μL with RNAse-free water.

### Quantitative real-time PCR

Genes were chosen for further analysis by PCR based on the analysis of next-generation sequencing data published previously [18]. These included the genes for the Toll-like receptors TLR2 and 4, the NOD-like receptor NLRC5, the chemokines CCL2, CXCL9, CXCL10 and CXCL11, as well as the antimicrobial peptides equine β-defensin 1 (EBD 1), lysozyme, secretory leukoprotease inhibitor (SLPI), secreted phospholipase A_2_ (sPLA_2_), lipocalin 2 (LCN 2), lactoferrin, uteroferrin and the uterocalin precursor (P19). In addition, seven genes with little variation were selected from the sequencing data: β-actin, glyceraldehyde-3-phosphate dehydrogenase (GAPDH) and the ribosomal proteins (RP) RPL17, RPL27A, RPL30, RPL32 and RPS5.

Primers used to detect the EBD 1 [45] and β-actin [17] genes were sourced from the literature, while specific primer pairs for all other genes of interest were designed using the NCBI nucleotide blast website [46]. Where possible, primer pairs spanned at least one intron to prevent amplification of contaminating genomic DNA (Table 1). The oligonucleotide sequences are shown in Table 1 and were commercially synthesised by Geneworks (Hindmarsh, SA, Australia).

**Table 1:** **Primer sequences used for quantitative reverse transcriptase PCR**

Gene name	Forward primer sequence	Reverse primer sequence	Insert size (bp)	Inter-exonic	Accession number/reference
GAPDH	GCTTCCCTTCCGCACTGCTA	CTCGGCCTTGACTGTGCCAT	222	Yes	NM_001163856.1
β-Actin	CGTGGGCCGCCCTAGGCACCA	TTGGCCTTAGGGTTCAGGGGGG	243	Yes	[17]
RPL17	TACAAAGTCATGCAAATCAAGAGGT	TTGGGAGCTTTGTTCACCTGG	339	Yes	NC_009151.2
RPL27A	AGGAAGACCCGGAAACTTCG	TTTGTAGTAGCCCGATCGCACC	315	Yes	NC_009150.2
RPL30	GGCCGTCCCGCACCTAAG	ATGACCAGTTTCGCTTTGCCT	166	Yes	XM_001491150.4
RPL32	TGGTCCACAATGTCAAGGAGC	TCGTCTATTCGTTTTCTTCGCTGC	180	No	XM_001500029.4
RPS5	TGCCATCATCAACAGTGGTCC	AGGTTTATTGGGGCTGTGGTCG	301	Yes	XM_001495360
TLR2	GTGGACGGTGTGGGTCTTAG	TGATGTCATTGGACCCCAGC	254	No	NM_001081796.1
TLR4	CTGTTACGGTGCGTCATGCT	ACCTGCAGTTCTGGGAAGTT	301	Yes	NM_001099769.1
NLRC5	CAGCTCCAGCACGGTATCAA	GCTAGTGTGGTCTTGCCCAT	377	Yes	XM_005608529.1
CCL2	CAACAACTCTCAGGCCGAA	ATCTCCTTGGCCAATATGGTCT	262	Yes	NM_001081931.1
CXCL9	AACAGTTTGCTCCAAGCCCT	CTTTTGACGAGGACGTTGCC	221	Yes	NM_001130078.1
CXCL10	CCTCTCTCTAGAACTGCACGC	GACGGTCTTGGACTCTGGATT	180	Yes	NM_001114940.1
CXCL11	GGCCCTGGAGTAAAAGCAGT	AACAGGCCGGAGAAAGTCAG	301	Yes	NM_001278930.1
EBD1	ATTTTCTCCTTGCCTTCCTCAT	GATACAAGTGCCGATCTGT	148	Yes	[45]
Lysozyme	TGGGTCTGTTTGGCCAGATG	GTGAGGTCGTGGTTCTGACA	284	Yes	XM_001494130.2
Lipocalin 2	GACCACAGCTACAACGTCAC	TCAGCTCCTTGGTCCTCCTAT	253	Yes	XM_005605817.1
Lactoferrin	TGGCTGAACTCCAAGGCAAA	GCGAGCATCACTCTCAGGAA	216	Yes	NM_001163974.1
Uteroferrin	CCAATATGGTCCATCGCGGA	GGGGTCCATGAAGTTCCCAG	180	Yes	NM_001246672
SLPI	CTTCCCCCTCGTGCTTCTTG	CAATGGGGTCCAGGCATTTG	209	Yes	XM_005604654.1
P19	TGATGACGGCTCACAAAACG	GCACCGATCAGTTTGGGTCAG	269	Yes	NM_001082509.2
sPLA2	GTTATGGCTTCTACGGTTGCCACT	ACACCCACGTTTCTGCAGACGATA	119	Yes	NM_001100113.2
TIMP1	CTACACCCCCGCTATGGAGA	CTGGTCCGTCCACAAGCAAT	268	Yes	NM_001082515.1

Standard curves were created to quantify absolute copy numbers for each gene. Briefly, positive PCR samples were pooled, DNA purified, a ligation reaction set up using the pGEM-T Easy Vector System (Promega) and α-Select Bronze Efficiency competent cells (Bioline, Alexandria, Australia) were transformed, inoculated onto LB agar and incubated overnight. A single colony was selected and cultured overnight in LB broth. Plasmid DNA was extracted from the overnight culture. After extraction, the plasmid was sent for sequencing to Monash Micromon (Melbourne, Australia) to confirm the sequence of the insert before dilution series were generated based on plasmid DNA concentration and size. Standard curves containing ten-fold dilutions from 30 000 000 to 300 copies per 5 μL were created for each gene.

Quantitative real-time PCRs were performed using the Rotor-Gene Q PCR machine (Qiagen). Each 20 μL reaction was set up with 10 μL iTaq Universal SYBR Green Supermix (Bio-Rad), 1 μL of each primer (forward and reverse, 10 μM), 3 μL RNAse-free water and 5 μL cDNA. A no-template control (RNAse-free water) and the appropriate standard curves were included in each run. All samples were run in triplicate. The reactions were incubated through an initial activation and denaturation step at 95 °C for 30 s, followed by 40 cycles of 5 s at 95 °C and 30 s at 60 °C. A melt curve analysis was performed between 65 and 95 °C in 0.5 °C increments to confirm the identity of the amplicons.

### Data analysis and statistical methods

All statistical analyses were performed using Stata release 14 (StataCorp, College Station, TX, USA). Results were considered statistically different if the value of *P* was <0.05. To assess the distribution of all variables, we generated frequency distributions for all categorical variables and histograms and summary measures for all continuous variables. For comparison of the presence of bacteria, a multi-level mixed-effects generalised linear model (with logit link function) was used with horse id entered as a random effect to account for repeated measurement and individual variability by horse. Data were log-transformed where appropriate.

Cycle stage comparisons were performed using interaction terms to combine time and cycle stage. The effect of the introduction of *E. coli* on clinical parameters and the expression of several genes associated with the immune response were analysed using a repeated measures ANOVA applying the Bonferroni correction for multiple comparisons. The interaction model was then used to estimate the fold change of gene expression at different time points in comparison to 0 h, which was designated as reference category.

## Results

### Clinical and gynaecological examination

All mares identified as being in the dioestrus phase of the cycle were found to have plasma progesterone concentrations between 25 and 33 nmol/L, as expected.

**Figure 2:**
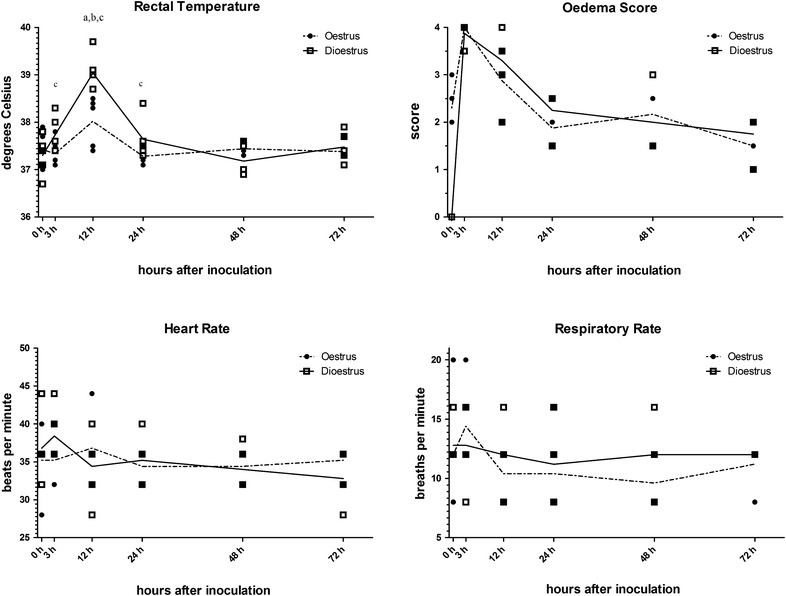
**Rectal temperature (°C), oedema score, heart rate (beats per minute) and respiratory rate (breaths per min).** Squares represent values for each of the five horses in dioestrus and circles for each horse in oestrus. The solid line is the mean in dioestrus and the dashed line the mean in oestrus. a, *P* < 0.05 compared to 0 h time point; b, *P* < 0.05 compared to previous time point; c, *P* < 0.05 compared to the alternative oestrous cycle stage at this time point.

The heart and respiratory rates of all mares remained within physiological limits throughout the experiment. Rectal temperatures were increased at 12 h pi compared to before and 3 h pi (*P* < 0.001; Figure 2). During dioestrus, the mares had higher rectal temperatures at 3, 12 and 24 h pi compared to their rectal temperatures after inoculation during oestrus at the same time points (*P* < 0.05). The rectal temperatures of the mares ranged between 38.7 and 39.7 °C at 12 pi in dioestrus, above the physiological range (37.5–38.5 °C). Temperatures were within the normal range in oestrus in the same mares at the same time point (Figure 2).

Oedema scores prior to inoculation with *E. coli* were in category 2 and 3 in oestrus, while no oedema was detected in dioestrus. Three hours after inoculation with *E. coli*, an oedema score of four was detected in all horses, irrespective of cycle stage. At 12 h pi, oedema scores ranged from 2 to 4 in both cycle stages, before decreasing to a range between 1 and 3 at 48 h pi and between 1 and 2 at 72 h pi (Figure 2).

### Bacteriological culture

No growth was detected prior to inoculation of *E. coli* in any of the cultures. Three hours afterwards, growth from biopsy sample cultures ranged from no to moderate growth during oestrus and from moderate to heavy growth in dioestrus, with more than 50 CFU/sample cultured from four of five mares (Table 2). From 12 h until 72 h pi, no growth was detected from any biopsies taken in oestrus, except for one horse with moderate growth of bacteria other than *E. coli* at 72 h pi. In dioestrus, the numbers of bacteria remained high, with all samples yielding moderate to heavy growth 12 h pi. In the period from 1 to 2 days after the introduction of *E. coli*, culture yielded no to moderate growth in dioestrus samples and no growth was detected in any cultures from 72 h samples. Cultures of swab samples yielded comparable results, with slightly higher numbers of bacteria detected (Table 2). Overall, 8.1 times more samples obtained in dioestrus were culture positive in comparison to oestrus (*P* = 0.008) and significantly more samples obtained between 3 and 24 h pi were culture positive in comparison to the baseline (0 h) (Additional file 1).

**Table 2:** **Bacterial growth from uterine biopsies and swabs before (0** **h) and 3, 12, 24, 48 and 72** **h after intrauterine inoculation of** ***E. coli***

Mare ID	Cycle	Biopsy sample						Swab sample					
		0 h	3 h	12 h	24 h	48 h	72 h	0 h	3 h	12 h	24 h	48 h	72 h
B	Oestrus	1	6	1	1^a^	0	2^a^	0	>50	12	0	0	0
E	Oestrus	0	0	1	4	1	0	0	>50	2	2	0	0
S	Oestrus	0	20	2	0	0	24^a^	1^a^	16	0	0	0	0
U	Oestrus	0	0	0	1^a^	0	3^a^	0	6	0	0	0	0
V	Oestrus	0	4	0	1^a^	0	2	0	3	1	0	>50^a^	0
B	Dioestrus	0	32	>50	16	8	0	0	>50	>50	0	18	0
E	Dioestrus	1^a^	>50	>50	7	0	0	0	>50	>50	>50	0	0
S	Dioestrus	0	>50	50	28	11	0	0	>50	>50	51	9	0
U	Dioestrus	0	>50	>50	8	4	0	0	>50	>50	43	14	2
V	Dioestrus	0	>50	15	2	3	0	0	>50	3	0	0	0

^a^Other uterine pathogen and/or contamination (lactose negative on MacConkey agar).

### Histopathology

**Figure 3:**
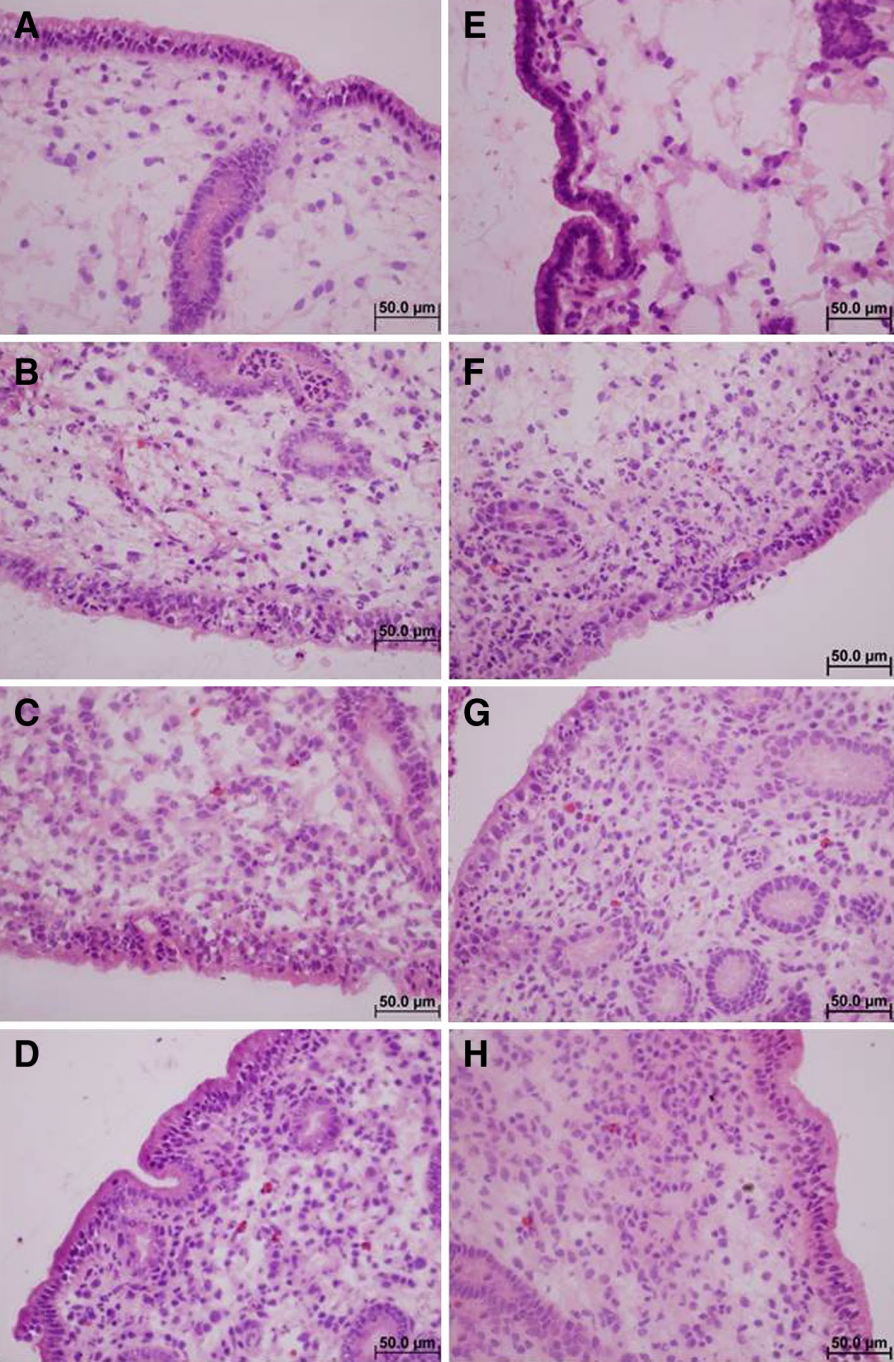
**Histology images of one horse.** Representative histology images of one horse obtained from biopsies taken in dioestrus (**A**–**D**), **A** before and at **B** 3 h, **C** 12 h and **D** 24 h after inoculation of *E. coli* and in oestrus (**E**–**F**), **E** before and at **F** 3 h, **G** 12 h and **H** 24 h after inoculation of *E. coli* at ×400 magnification.

One sample taken in dioestrus at 24 h was missing and omitted from the analysis. No inflammation was detected prior to the introduction of *E. coli* in any of the samples. Three hours afterwards, the level of neutrophilia ranged from moderate to severe in dioestrus samples, while all oestrus samples had very severe levels of neutrophilia. After 12 h, samples in both cycle stages showed signs of severe to very severe neutrophilia. At 24 h, oestrus samples were classified as having moderate to severe neutrophilia and dioestrus samples moderate to very severe neutrophilia, with more neutrophils present in all dioestrus samples when paired with the oestrus samples from the same time point in the same horse. Neutrophils were detected in the epithelium of most samples within the first 12 h, regardless of cycle stage, while there were fewer seen in oestrus at 24 h pi in two of five samples. All but one 3 h sample in dioestrus had neutrophils inside endometrial glands, while two oestrus samples did not have any neutrophils in this location 12 h pi with *E. coli*. Eosinophil counts ranged from 0 to 16 at 3 h, 1 to 56 at 12 h and 0 to 33 at 24 h (Table 3; Figure 3).

**Table 3:** **Leukocyte counts (neutrophils and eosinophils) before (0** **h) and 3, 12 and 24** **h after intrauterine inoculation of** ***E. coli***

Mare ID	Cycle	Neutrophils				Eosinophils			
		0 h	3 h	12 h	24 h	0 h	3 h	12 h	24 h
B	Oestrus	7 (Ep1, G0)	>150 (Ep3, G1)	110 (Ep2, G0)	60 (Ep1, G0)	2	10	1	0
E	Oestrus	1 (Ep0, G0)	>150 (Ep3, G2)	110 (Ep3, G0)	75 (Ep2, G0)	3	15	10	22
S	Oestrus	7 (Ep0, G0)	>150 (Ep3, G1)	>150 (Ep3, G3)	80 (Ep3, G2)	0	16	1	4
U	Oestrus	5 (Ep0, G0)	>150 (Ep3, G3)	>150 (Ep3, G1)	130 (Ep3, G0)	1	14	23	8
V	Oestrus	8 (Ep1, G0)	>150 (Ep3, G2)	>150 (Ep3, G2)	130 (Ep3, G3)	1	16	2	1
B	Dioestrus	2 (Ep0, G0)	80 (Ep1, G0)	120 (Ep3, G1)	90 (Ep3, G0)	0	0	56	7
E	Dioestrus	4 (Ep0, G0)	125 (Ep3, G3)	140 (Ep3, G2)	140 (Ep3, G0)	1	6	15	8
S	Dioestrus	7 (Ep0, G0)	130 (Ep3, G1)	>150 (Ep3, G3)	>150 (Ep3, G2)	2	0	35	28
U	Dioestrus	6 (Ep2, G0)	145 (Ep3, G3)	>150 (Ep3, G3)		0	14	21	
V	Dioestrus	7 (Ep0, G1)	145 (Ep3, G3)	>150 (Ep3, G3)	>150 (Ep3, G3)	4	6	14	33

Ep, presence of neutrophils in epithelium in 1, 2 or 3 fields.

G, presence of neutrophils in endometrial glands in 1, 2 or 3 field.

### Endometrial gene expression

Seven genes were tested to identify the most stable genes under the experimental conditions used for the study. Of these, GAPDH, RPL30, RPL32 and RPS5 showed no significant variation between any of the time points or cycle stages. This also confirmed that the efficiency of the cDNA synthesis was consistent between samples, allowing for gene-specific absolute quantification.

The target genes were divided into several groups based on their function in the immune system. Statistical results for gene expression levels can be found in Additional file 2.

### Pathogen recognition receptors

**Figure 4:**
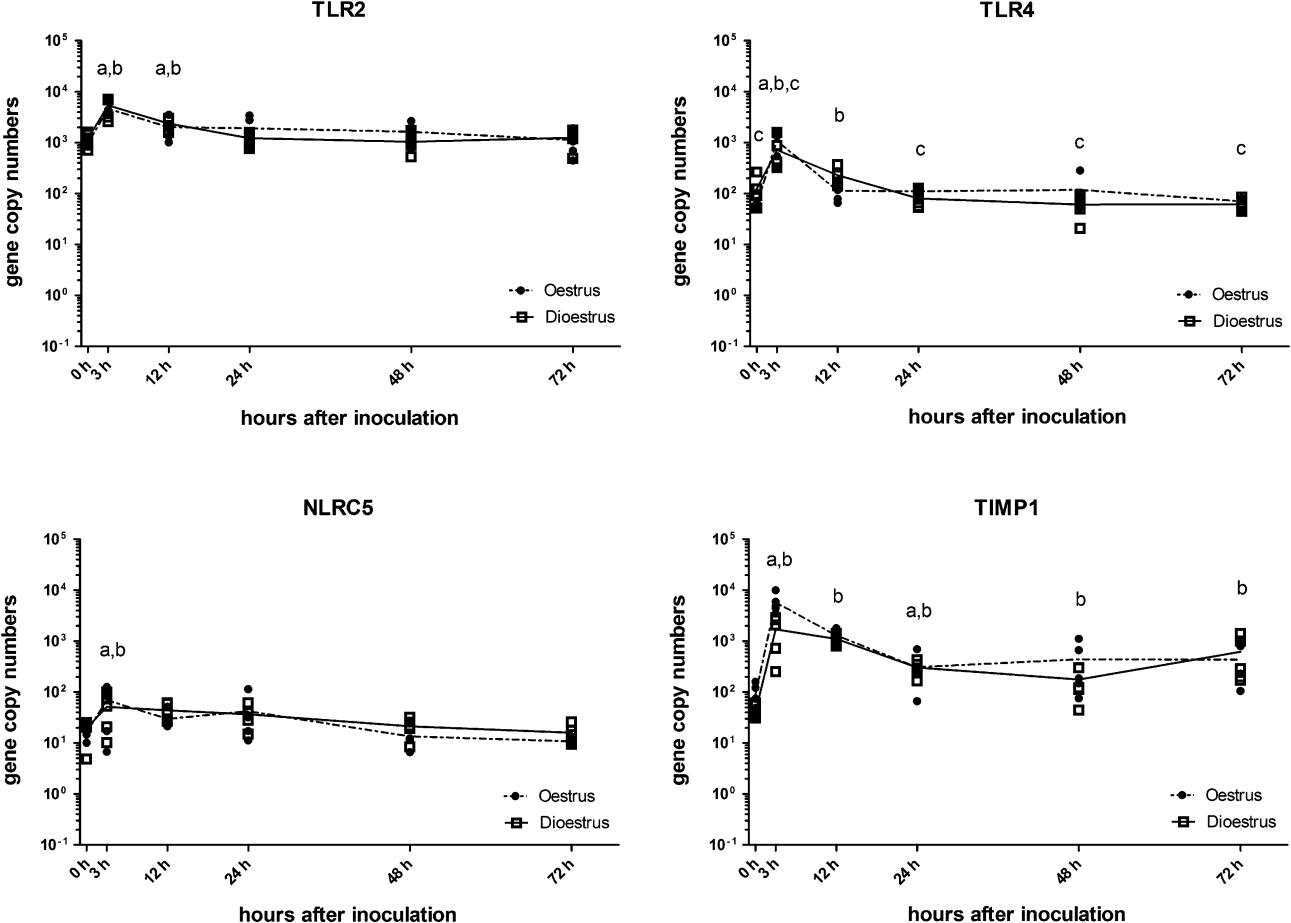
**Levels of mRNA transcripts of endometrial TLR2, TLR4, NLRC5 and TIMP1 genes.** Gene expression was measured in copy numbers/ng RNA determined from standard curves for each gene. Squares indicate values for each of the five horses in dioestrus and circles for each horse in oestrus. The solid line is the mean in dioestrus and the dashed line the mean in oestrus. a, *P* < 0.05 compared to 0 h time point; b, *P* < 0.05 compared to previous time point; c, *P* < 0.05 compared to the alternative oestrus cycle stage at this time point.

The mRNA expression levels of the genes for the pathogen recognition receptors TLR2, TLR4 and NLRC5 were two to four times higher at 3 h pi compared to levels before inoculation. The levels of expression of these genes decreased to baseline between 12 and 24 h pi (Table 4; Figure 4).

**Table 4:** **Linear mixed regression coefficients and confidence intervals for all statistically significantly different comparisons between time points after inoculation with** ***E. coli*** **and baseline (0** **h) for pathogen recognition receptors, chemokines and tissue inhibitor of metallopeptidase 1**

Group	Gene	Time (h)	*P* value	Contrast	(95% CI)
Pathogen recognition receptors	TLR2	3	<0.001	4.3	(2.7, 7)
		12	0.005	2.0	(1.2, 3.2)
	TLR4	3	<0.001	5.5	(3.4, 9.1)
		12	0.006	2.0	(1.2, 3.3)
		48	0.007	0.5	(0.3, 0.8)
		72	0.022	0.6	(0.3, 0.9)
	NLRC5	3	0.018	2.3	(1.2, 4.5)
		12	0.010	2.5	(1.2, 4.9)
Chemokines	CCL2	3	<0.001	257.3	(126.2, 524.3)
		12	<0.001	13.9	(6.8, 28.3)
		24	<0.001	4.4	(2.2, 9)
		48	0.001	3.3	(1.6, 6.8)
	CXCL9	3	<0.001	25.5	(12.6, 51.5)
		12	<0.001	126.6	(62.7, 255.6)
		24	<0.001	41.0	(20.3, 82.8)
		48	<0.001	6.6	(3.3, 13.4)
	CXCL10	3	<0.001	442.7	(189.2, 1035.4)
		12	<0.001	228.1	(97.5, 533.5)
		24	<0.001	17.2	(7.4, 40.2)
		48	<0.001	4.6	(2, 10.9)
	CXCL11	3	<0.001	116.4	(45.1, 301)
		12	<0.001	388.7	(150.4, 1004.6)
		24	<0.001	23.0	(8.9, 59.5)
		48	<0.001	8.5	(3.3, 22)
Tissue inhibitor of matrix metallo-peptidases	TIMP1	3	<0.001	31.7	(14.1, 71.5)
		12	<0.001	27.8	(12.4, 62.7)
		24	<0.001	7.4	(3.3, 16.7)
		48	0.002	3.6	(1.6, 8.2)
		72	<0.001	10.9	(4.9, 24.6)

Contrasts represent back-transformed regression coefficients for multiplicative interpretation. Time, time point in hours (h) after inoculation of *E. coli.*

CI: confidence interval.

### Chemokines

**Figure 5:**
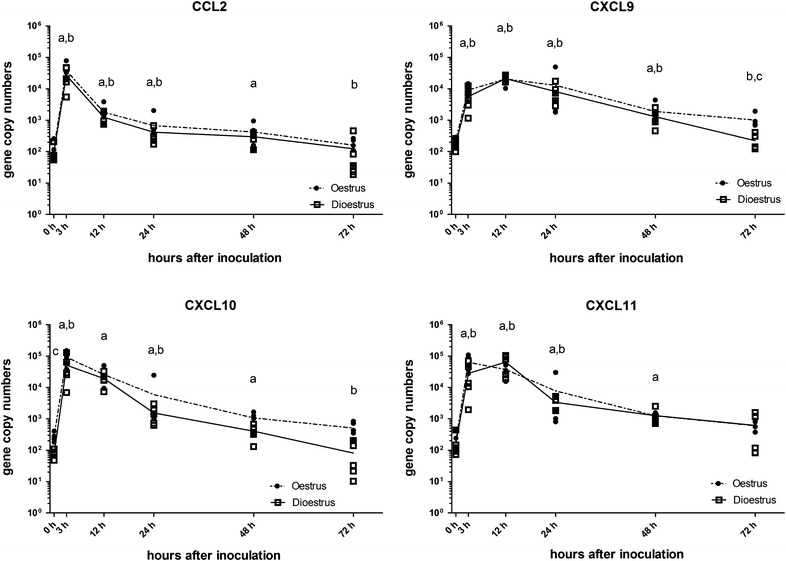
**Levels of mRNA transcripts of endometrial CCL2, CXCL9, CXCL10 and CXCL11 genes.** Gene expression was measured in copy numbers/ng RNA determined from standard curves for each gene. Squares indicate values for each of the five horses in dioestrus and circles for each horse in oestrus. The solid line is the mean in dioestrus and the dashed line the mean in oestrus. a, *P* < 0.05 compared to 0 h time point; b, *P* < 0.05 compared to previous time point; c, *P* < 0.05 compared to the alternative oestrus cycle stage at this time point.

There was an increase in expression of all four chemokine genes analysed at 3 h pi, with levels starting to decrease between 12 and 24 h pi and reaching pre-inoculation levels at 72 h pi. Peak expression levels ranged between 126 and 443 times that of the respective baseline levels and were reached at 3 h pi for CCL2, CXCL10 and CXCL11 and at 12 h pi for CXCL9 (Table 4; Figure 5).

### Tissue inhibitors of metallopeptidases

The levels of expression of the gene for the metallopeptidase inhibitor TIMP-1 increased to 32 times baseline levels at 3 h after inoculation of *E. coli* and remained elevated throughout the experimental period (Table 4; Figure 4).

### Antimicrobial peptides

**Figure 6:**
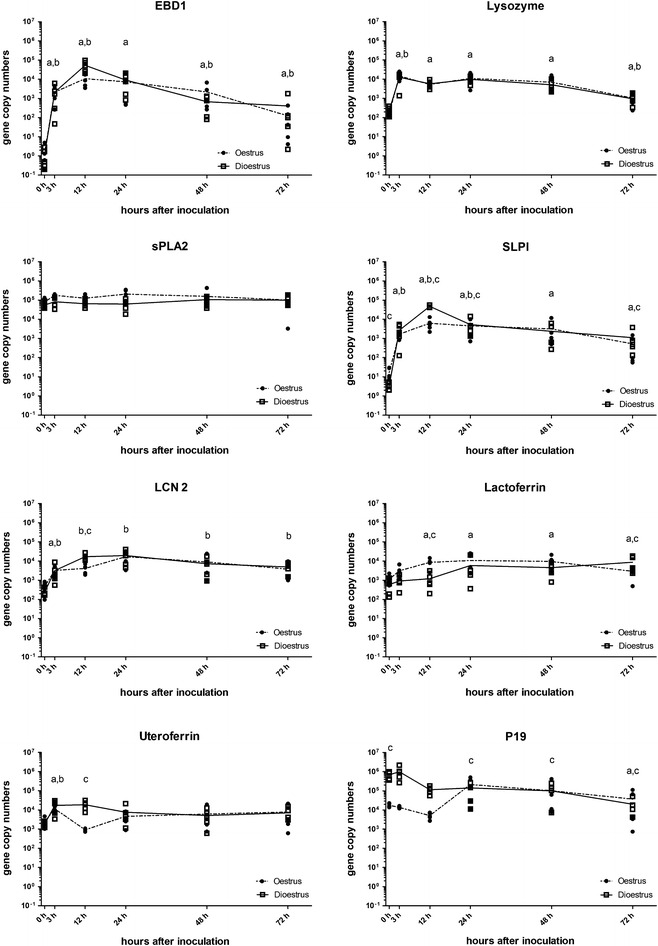
**Levels of mRNA transcripts of EBD1, lysozyme, sPLA2, SLPI, LCN2, lactoferrin, uteroferrin and P19 uterocalin genes.** Gene expression was measured in copy numbers/ng RNA determined from standard curves for each gene. Squares indicate values for each of the five horses in dioestrus and circles for each horse in oestrus. The solid line is the mean in dioestrus and the dashed line the mean in oestrus. a, *P* < 0.05 compared to 0 h time point; b, *P* < 0.05 compared to previous time point; c, *P* < 0.05 compared to the alternative oestrus cycle stage at this time point.

Expression profiles of genes for antimicrobial peptides exhibited greater variability in response to *E. coli* (Table 5; Figure 6). While most genes were expressed at higher levels between 3 and 12 h after inoculation compared to baseline, expression of P19 and sPLA_2_ remained unchanged. The greatest difference between oestrus and dioestrus expression profiles was seen at 12 h pi. Genes for LCN2, UFN and SLPI were expressed at higher levels in dioestrus, while the gene for LFN was expressed at higher levels in oestrus at this time point.

**Table 5:** **Linear mixed regression coefficients and confidence intervals for all statistically significantly different comparisons between time points after inoculation with** ***E. coli*** **and baseline (0** **h) for antimicrobial peptides**

Group	Gene	Time (h)	*P* value	Contrast	(95% CI)
Antimicrobial peptides	EBD1	3	<0.001	1185.8	(239.8, 5863)
		12	<0.001	65950.5	(13 339, 326 071.6)
		24	<0.001	7309.0	(1478.3, 36 137.1)
		48	<0.001	580.3	(117.4, 2869.3)
		72	<0.001	102.3	(20.7, 505.6)
	Lysozyme	3	<0.001	41.5	(19.8, 87)
		12	<0.001	23.6	(11.3, 49.4)
		24	<0.001	40.1	(19.2, 84.1)
		48	<0.001	19.4	(9.3, 40.7)
		72	0.001	3.6	(1.7, 7.5)
	SLPI	3	<0.001	510.8	(173.1, 1507.9)
		12	<0.001	15,661.6	(5305.8, 46 229.3)
		24	<0.001	1283.8	(434.9, 3789.6)
		48	<0.001	408.6	(138.4, 1206)
		72	<0.001	197.9	(67, 584.1)
	LCN2	3	<0.001	9.4	(3.7, 23.8)
		12	<0.001	71.6	(28.2, 182.2)
		24	<0.001	64.4	(25.3, 163.8)
		48	<0.001	20.4	(8, 51.9)
		72	<0.001	19.3	(7.6, 49)
	LFN	24	<0.001	6.3	(2.7, 15.1)
		48	<0.001	8.1	(3.4, 19.4)
		72	<0.001	14.7	(6.2, 34.9)
	UFN	3	<0.001	7.2	(2.8, 18.6)
		12	<0.001	9.2	(3.6, 23.7)
		24	0.035	2.8	(1.1, 7.1)
		72	0.014	3.3	(1.3, 8.4)
	P19	12	0.006	0.2	(0, 0.6)
		24	0.002	0.1	(0, 0.5)
		48	<0.001	0.1	(0, 0.3)
		72	<0.001	0.0	(0, 0.1)

Contrasts represent back-transformed regression coefficients for multiplicative interpretation.

Time, time point in hours (h) after inoculation of *E. coli.*

CI: confidence interval.

EBD-1 gene expression did not differ between cycle stages. There was a very rapid increase from levels of between 0 and 5 copies/ng of RNA prior to inoculation of *E. coli* to levels of between 3488 and 96 657 copies/ng of RNA at 12 h after inoculation.

The expression of the lysozyme gene was increased to 42 times baseline at 3 h pi and remained at a similar level until 24 h pi, after which levels decreased.

No significant difference in expression was detected for the sPLA_2_ gene either over time or between cycle stages, with levels averaging 109 409 copies/ng of RNA.

SLPI gene expression increased to more than 15 000 times baseline at 12 h pi, before slowly decreasing. Gene expression started with higher levels in oestrus compared to dioestrus, but dioestrus levels were higher than oestrus levels at 12 and 24 h pi. At 48 h pi the level of expression was similar in both oestrus and dioestrus samples, but oestrus levels were higher at 72 h pi.

Expression of the LCN2 gene increased to 72 times baseline levels at 12 h pi, before slowly declining. The levels of expression were higher in dioestrus at 12 h pi, compared to in oestrus.

The increase in gene expression of the LFN gene was continuous, reaching 14.7 times baseline levels at 72 h pi. While LFN gene expression in oestrus was higher throughout the first 48 h pi, dioestrus levels were higher at 72 h pi.

UFN gene expression increased to 7.2 times higher than baseline levels at 3 h pi in both cycle stages. At 12 h after the introduction of *E. coli*, expression remained at similar levels in dioestrus samples. By 24 h, oestrus and dioestrus levels of mRNA for the UFN gene were similar and did not differ from those in baseline samples.

Expression of the P19 gene did not change over the first 48 h pi, but then decreased to levels 43 times lower than baseline. Dioestrus levels higher before and at 3 and 12 h pi.

## Discussion

Many broodmares are affected by persistent endometritis, a disease that has been identified as the third most common medical problem in American horses [47], and results in reduced pregnancy rates [2]. Both breeding and the introduction of bacteria have been shown to induce an inflammatory response in the equine uterus [14, 42, 48]. Thus, the first step to understanding the pathophysiological mechanisms underlying the diseases is to analyse the precise timeline of the inflammatory response in the equine uterus during infectious endometritis in healthy mares. In addition, the evaluation of the impact the different hormonal environments associated with the oestrous cycle have on this process, may provide further insights into the use of experimental inoculation with *E. coli* as a model of infectious endometritis. As the uterine bacterial load during PMIE is unknown, a relatively high dose was used in this experiment in order to reliably induce a measurable immune response in mares resistant to PMIE [7]. The bacterial growth detected during these studies showed that infection was reliably induced in both cycle stages and that it was cleared within the 72 h study period (Table 2), suggesting an appropriate dose of *E. coli* was used. The clinical and gynaecological examinations (Figure 2), as well as the histological cell counts (Figure 3; Table 3), confirmed that transient inflammation was induced in all horses. The combination of a significant increase in body temperature 12 h pi, inflammatory uterine oedema at 3 h pi and a rapid influx of neutrophils within the first 3 h pi were clearly indicative of this. Similarly, the expression of most of the analysed genes associated with the innate immune system was up-regulated between 3 and 12 h pi.

It was assumed that the effect of collection of repeated biopsies in the same cycle was negligible compared to the strong reaction to *E. coli*, particularly as previous studies have detected no effect of repeated biopsies on expression of a range of cytokines [7]. The fact that monocultures of *E. coli* were detected in most of the cultured biopsy and swab samples renders a contamination of the uterus with additional bacteria unlikely as these introduced bacteria would have reached detectable levels by the next sample collection. While we cannot entirely exclude an inflammatory effect of the repeated tissue disruption, most gene expression levels in this study returned to baseline levels within 24–72 h pi despite repeated biopsy collection. Alternatively, samples could have been collected in different cycles, which would have introduced more variability regarding individual changes throughout the breeding season, or from different horses, which would have increased the bias from biological inter-individual variations.

Our previous next-generation sequencing analysis revealed that a large number of immune response genes were up-regulated within the first 3 h after the introduction of *E. coli* into the uterus [18]. To investigate the progression of the inflammatory response after this time point, expression of a panel of genes was followed by qPCR until 72 h after inoculation. This revealed that expression of most of the analysed innate immune response genes increased very rapidly in response to the introduction of bacteria, but that levels declined to baseline or nearly baseline within 48–72 h. The mRNA expression profiles for the initial 3 h pi provide very similar results to our previous deep-sequencing analysis validating both techniques [18].

### Pathogen recognition receptors

When *E. coli* enters the body, its LPS is detected by PRR, particularly TLR4 [20, 21]. Our results showed that transcripts of the TLR4 gene were present in the uterus of all five mares in both stages of the oestrous cycle prior to inoculation. The introduction of *E. coli* resulted in a significant increase in gene expression by 3 h pi, but expression returned to baseline levels by 12 h pi. As activation of cells through TLR4 ultimately leads to an increase in cytokine production [20] and prolonged inflammation is undesirable in the uterus, this very rapid, but short-lived, up-regulation in mRNA levels at the initial point of detection is likely to be necessary for effective elimination of bacteria. Similarly, the expression of the TLR2 gene, which has a supportive role in detection of LPS [22], is only up-regulated for the first 12 h pi. The function of NLRC5 has been described as inhibiting pro-inflammatory cytokine production [49], but also as inducing their expression through the inflammasome complex [50]. We found that the expression of the NLRC5 gene was slightly up-regulated 3 h after the introduction of *E. coli*, suggesting a pro-inflammatory role. All three PRR genes had very similar patterns of expression over time, with the TLR4 gene showing the greatest increase in response to *E. coli* (Figure 4). Surprisingly, the expression of the PRR genes in response to *E. coli* did not differ greatly between oestrus and dioestrus.

### Chemokines

Chemokines are produced by sentinel cells, such as macrophages, in response to activation through PRRs on their cell surface. They are a subfamily of cytokines recruiting various leucocytes, such as monocytes by CCL2, and T lymphocytes by CXCL9, 10 and 11 [51–54]. In addition, CXCL9, 10 and 11 have been suggested to have antimicrobial activities [25, 26, 55]. We found that the mRNA levels of all analysed chemokines were elevated as early as 3 h after the introduction of *E. coli* into the uterus, reaching a peak expression levels between 3 and 12 h after infection and only decreasing to baseline levels at 72 h pi (Figure 5). CCL2 gene expression decreased already between 3 and 12 h, while levels of expression of the CXCL9, 10 and 11 genes were still high at 12 h pi. CCL2 was also the only chemokine analysed that has no direct antimicrobial effect on *E. coli*, with the other three CXCL chemokines able to kill 60–80% of cultured *E. coli* [26]. Thus, a longer up-regulation of CXCL9, 10 and 11 may be more beneficial in aiding the clearance of *E. coli* from the uterus.

### Tissue inhibitor of metallopeptidases

TIMP1 regulates the expression of MMPs, thus indirectly having an inhibitory effect on the immune response. It has been shown that TIMP1 levels are reduced in human decidua prior to parturition, resulting in increased MMP levels and increased cytokine release [56, 57]. In the equine uterus, MMPs have only been investigated for the potential role in periglandular fibrosis and endometriosis [58]. Our next-generation sequencing study was the first to associate MMP gene expression with uterine inflammation in horses and also the first to detect TIMP1 gene expression in the equine uterus [18]. Its expression reached peak levels 3 h pi (Figure 4). This may suggest that mechanisms for the containment of the inflammatory process are already prepared 3 h after the introduction of *E. coli* into the uterine environment. Interestingly, levels of expression remained elevated throughout the study period in both cycle stages, suggesting a continuous suppressive influence throughout the inflammatory process irrespective of the stage of the oestrus cycle.

### Antimicrobial peptides

AMP are small peptides of less than 100 amino acids that have various antimicrobial actions and have been detected in multiple organs in the horse [27]. One bactericidal mechanism is to destabilise the bacterial cell membrane either by formation of pores in the lipid bilayer or by enzymatic degradation of the phospholipids [28]. Similar mechanisms are used by lysozyme, sPLA_2_ and probably EBD1. Both human β-defensins and equine lysozyme have been shown to have bactericidal effects on Gram-negative bacteria [59, 60]. No study has evaluated the antimicrobial potency or mode of action of EBD1. In contrast, sPLA_2_, the gene for which was analysed in this study and which is in Group2A of the phospholipases (PLA2G2A) has no bactericidal activity against Gram-negative bacteria, including *E. coli* [61]. We found that expression of the EBD1 gene was more than 65 000 times higher at 12 h after infection compared to baseline levels. Lysozyme gene expression only increased 42 times within the first 3 h pi. In contrast, sPLA_2_ was constitutively expressed at high levels of around 100 000 copies/ng of RNA (Figure 6). A previous study has detected high levels of sPLA_2_ in the uterus of non-pregnant mares, with lower levels of expression seen in normal pregnant mares and increasing levels, similar to those detected in non-pregnant mares, during induced abortions [62]. The great variance in gene expression profiles within this group of AMP suggests that the uterine immune system can react in a specific and finely-tuned manner to the introduction of bacteria. While the EBD1 gene had by far the greatest increase in expression, its post inoculation expression levels were comparable to those of lysozyme and sPLA_2_. In contrast, sPLA_2_ levels did not increase, but were already high prior to the introduction of *E. coli*. Hence, up-regulation of EBD-1 gene expression in response to the introduction of *E. coli* may only be necessary because it is expressed at such low levels prior to stimulation. Expression of the sPLA_2_ gene may not be stimulated by *E. coli* because Gram-negative bacteria are not the primary targets of its antimicrobial activity. The stimulation of expression of the EBD1 gene by *E. coli* may suggest that EBD1 has antimicrobial activity against this pathogen.

SPLI has bactericidal activity against both Gram-positive and Gram-negative bacteria, selectively inhibiting serine proteases of the subtilisin superfamily, which are uniquely expressed by fungi and bacteria [32]. Expression of the SPLI gene had increased to more than 15 000 times that of baseline levels by 12 h after the introduction of *E. coli*. SLPI gene transcription was induced by *E. coli* during both cycle stages, but at greater levels during dioestrus. One possible explanation for this is that the adaptive immune response has to be down-regulated in order to protect a potential half-allogenic conceptus from being attacked by the immune response. Hence, the innate immune response, or parts thereof, have to be more alert to compensate for this and still allow successful prevention of any possible infections.

Finally, LCN2, LFN, UFN and P19 have all been shown to bind elements such as iron, either as part of the immune response or in a trans-placental transporter function [33, 34, 37, 38]. In this study, the LCN2 and UFN genes had relatively similar expression profiles, reaching peak expression levels 12 h pi and with higher levels reached at this time point in dioestrus than in oestrus. In contrast, the expression of the LFN gene increased slowly and continuously, with levels 15 times higher than the baseline at 72 h pi and oestrus levels being higher than dioestrus levels at 12 h pi. The P19 uterocalin gene was initially expressed at levels 37 times higher in dioestrus than in oestrus, but this difference was eliminated by 24 h pi (Figure 6). The bacteriostatic effect of these binding elements may explain the slower response and the lesser magnitude of the increase in expression of this group of AMP genes compared to those of some of the other genes that encode bactericidal factors. LCN2 has been suggested previously as a marker for the response to tissue injuries or insults, including the introduction of PAMPs [63]. While uteroferrin has not been described previously as part of the uterine immune arsenal, the significant up-regulation of its gene in response to infection with *E. coli* suggests that it may play a role. Contrarily, expression of the P19 gene does not seem to be affected by infection with *E. coli* in dioestrus, but in oestrus increases by 24 h pi (Figure 6).

Overall, most of the AMP genes we investigated were expressed at higher levels within the first 3–12 h pi. While the cycle stage seemed to have only a small overall effect on the response to *E. coli*, there seemed to be some variations in the response at the 12 h time point (Figure 6).

## Additional files

**Additional file 1.** **Mixed-effects logistic regression model for bacteria growth.** Mixed-effects logistic regression model for bacteria growth in cultured biopsies obtained in oestrus and dioestrus before and at 3, 12, 24, 48 and 72 h after inoculation with *E. coli.*

**Additional file 2.** **Statistical results for gene expression levels of all analysed genes.** ANOVA, Bonferroni and interaction model results for the pathogen recognition receptors TLR2, TLR4, NLRC5, the chemokines CCL2, CXCL9, CXCL10, CXCL11, the tissue inhibitor of metallopeptidases (TIMP1) and the antimicrobial peptides lysozyme, lipocalin (LCN2), lactoferrin, uteroferrin, secretory leukoprotease inhibitor (SLPI), uterocalin P19, secreted phospholipase A_2_ (sPLA2) and equine β-defensin 1 (EBD1).

## Abbreviations

CFU: colony forming units
*E. coli*: *Escherichia coli* (*E. coli*)
LCN2: lipocalin 2
LFN: lactoferrin
LPS: lipopolysaccharide
MMP: matrix metalloproteinases
NLR: nucleotide-binding oligomerisation domain (NOD) receptor
P19: uterocalin P19
PAMP: pathogen-associated molecular pattern
PMIE: persistent mating-induced endometritis
*S. equi* ss *zooepidemicus*: *Streptococcus equi* subspecies *zooepidemicus*
SLPI: secretory leukoprotease inhibitor
sPLA_2_: secreted phospholipase A_2_
TIMP: tissue inhibitor of metalloproteinases
TLR: Toll-like receptor
UFN: uteroferrin
