# Supplementary material for: Oestrous cycle-dependent equine uterine immune response to induced infectious endometritis
Source: Vet Res. 2016 Nov 8;47:110. doi: 10.1186/s13567-016-0398-x (PMC5101692; doi:10.1186/s13567-016-0398-x)
Supplement: Supplementary file 1 — Additional file 1. Mixed-effects logistic regression model for bacteria growth. Mixed-effects logistic regression model for bacteria growth in cultured biopsies obtained in oestrus and dioestrus before and at 3, 12, 24, 48 and 72 h after inoculation with E. coli. [file 13567_2016_398_MOESM1_ESM.docx]

**Additional file 1 Mixed-effects logistic regression model for bacteria growth in cultured biopsies obtained in oestrus and dioestrus before and at 3, 12, 24, 48 and 72 h after inoculation with *E. coli*.**

| **Variable** | ***β*** | **SE(*β*)** | **OR** | **95% CI of OR** | ***P*-value** |
| --- | --- | --- | --- | --- | --- |
| Constant | -2.85 | 0.03 | — | — | — |
|  |  |  |  |  |  |
| cycle  (D vs E) | 2.64 | 6.50 | 8.17 | [1.7, 38.9] | 0.008 |
|  |  |  |  |  |  |
| 3 h | 2.93 | 110.07 | 74.74 | [4.2, 1340.3] | 0.003 |
| 12 h | 2.93 | 110.07 | 74.74 | [4.2, 1340.3] | 0.003 |
| 24 h | 2.29 | 31.12 | 22.81 | [1.6, 330.6] | 0.022 |
| 48 h | 1.95 | 18.04 | 13.50 | [1, 185.2] | 0.051 |
| 72 h | 0 | 1.54 | 1.00 | [0, 20.4] | 1 |
|  |  |  |  |  |  |
| **Random effect term** | **Var** | **SE(Var)** |  |  |  |
| horseid | 1.06E-16 | 5.63E-33 | — | — | — |

*N* = 60, Log likelihood= -25.79; d.f. = 6; *P* = 0.013; AIC = 65.58255.

Reference category is designated to oestrus 0 h.
SE = Standard Error, OR = Odds Ratio, CI = Confidence Interval, Var =Variance.
